# ChatGPT and the Future of Digital Health: A Study on Healthcare Workers’ Perceptions and Expectations

**DOI:** 10.3390/healthcare11131812

**Published:** 2023-06-21

**Authors:** Mohamad-Hani Temsah, Fadi Aljamaan, Khalid H. Malki, Khalid Alhasan, Ibraheem Altamimi, Razan Aljarbou, Faisal Bazuhair, Abdulmajeed Alsubaihin, Naif Abdulmajeed, Fatimah S. Alshahrani, Reem Temsah, Turki Alshahrani, Lama Al-Eyadhy, Serin Mohammed Alkhateeb, Basema Saddik, Rabih Halwani, Amr Jamal, Jaffar A. Al-Tawfiq, Ayman Al-Eyadhy

**Affiliations:** 1 College of Medicine, King Saud University, Riyadh 11587, Saudi Arabia; 2 Pediatric Department, King Saud University Medical City, King Saud University, Riyadh 11411, Saudi Arabia; 3 Evidence-Based Health Care & Knowledge Translation Research Chair, King Saud University, Riyadh 11587, Saudi Arabia; 4 Critical Care Department, King Saud University Medical City, Riyadh 11411, Saudi Arabia; 5 Research Chair of Voice, Swallowing, and Communication Disorders, ENT Department, College of Medicine, King Saud University, Riyadh 11587, Saudi Arabia; 6 Solid Organ Transplant Center of Excellence, King Faisal Specialist Hospital and Research Center, Riyadh 11564, Saudi Arabia; 7 Pediatric Nephrology Department, Prince Sultan Military Medical City, Riyadh 12233, Saudi Arabia; 8 Division of Infectious Diseases, Department of Internal Medicine, College of Medicine, King Saud University, Riyadh 11451, Saudi Arabia; 9 College of Pharmacy, Alfaisal University, Riyadh 11533, Saudi Arabia; 10 College of Medicine, Jordan University of Science and Technology, Irbid 22110, Jordan; 11 Sharjah Institute of Medical Research, University of Sharjah, Sharjah 27272, United Arab Emirates; 12 Department of Community and Family Medicine, College of Medicine, University of Sharjah, Sharjah 27272, United Arab Emirates; 13 School of Population Health, Faculty of Medicine & Health, UNSW Sydney, Sydney, NSW 2052, Australia; 14 Department of Clinical Sciences, College of Medicine, University of Sharjah, Sharjah 27272, United Arab Emirates; 15 Department of Family and Community Medicine, King Saud University Medical City, Riyadh 11411, Saudi Arabia; 16 Specialty Internal Medicine and Quality Department, Johns Hopkins Aramco Healthcare, Dhahran 34465, Saudi Arabia; 17 Infectious Disease Division, Department of Medicine, Indiana University School of Medicine, Indianapolis, IN 46202, USA; 18 Infectious Disease Division, Department of Medicine, Johns Hopkins University School of Medicine, Baltimore, MD 21218, USA

**Keywords:** ChatGPT, artificial intelligence, healthcare workers, perception, AI chatbots, medicolegal implications, credibility

## Abstract

This study aimed to assess the knowledge, attitudes, and intended practices of healthcare workers (HCWs) in Saudi Arabia towards ChatGPT, an artificial intelligence (AI) Chatbot, within the first three months after its launch. We also aimed to identify potential barriers to AI Chatbot adoption among healthcare professionals. A cross-sectional survey was conducted among 1057 HCWs in Saudi Arabia, distributed electronically via social media channels from 21 February to 6 March 2023. The survey evaluated HCWs’ familiarity with ChatGPT-3.5, their satisfaction, intended future use, and perceived usefulness in healthcare practice. Of the respondents, 18.4% had used ChatGPT for healthcare purposes, while 84.1% of non-users expressed interest in utilizing AI Chatbots in the future. Most participants (75.1%) were comfortable with incorporating ChatGPT into their healthcare practice. HCWs perceived the Chatbot to be useful in various aspects of healthcare, such as medical decision-making (39.5%), patient and family support (44.7%), medical literature appraisal (48.5%), and medical research assistance (65.9%). A majority (76.7%) believed ChatGPT could positively impact the future of healthcare systems. Nevertheless, concerns about credibility and the source of information provided by AI Chatbots (46.9%) were identified as the main barriers. Although HCWs recognize ChatGPT as a valuable addition to digital health in the early stages of adoption, addressing concerns regarding accuracy, reliability, and medicolegal implications is crucial. Therefore, due to their unreliability, the current forms of ChatGPT and other Chatbots should not be used for diagnostic or treatment purposes without human expert oversight. Ensuring the trustworthiness and dependability of AI Chatbots is essential for successful implementation in healthcare settings. Future research should focus on evaluating the clinical outcomes of ChatGPT and benchmarking its performance against other AI Chatbots.

## 1. Introduction

Artificial Intelligence (AI) is the ability of digital computers or computer-controlled robots to perform tasks typically associated with intelligent beings [1,2]. Chatbots are platforms that emulate human interaction through written, vocal, and visual communication modes. OpenAI launched Chat Generative Pre-trained Transformer version 3.5 (ChatGPT-3.5) on 30 November 2022, as an advanced AI Chatbot. Early research highlighted both potentially promising and concerning healthcare applications for ChatGPT [3,4]. As an advanced large language model (LLM), ChatGPT has shown potential in various medical applications, such as identifying research topics, assisting in clinical and laboratory diagnosis, and providing updates and new developments to healthcare professionals. It also held promise in the development of virtual assistants to aid patients in managing their health [5]. Furthermore, ChatGPT had been looked at to augment the response to pandemics or the integration with the global burden of disease to come up with a model to help in clinical and translational medicine [6,7].

On the other hand, the use of ChatGPT and similar AI Chatbots in healthcare also raises ethical and legal concerns, including potential copyright infringement, medicolegal complications, and the need for transparency in AI-generated content [5,8]. Evaluating AI’s accuracy in providing medical information and its ability to provide verified data for patients and healthcare workers is crucial [9]. 

With ChatGPT’s recent emergence, there is no available data to assess healthcare workers’ (HCWs) experience, which is crucial information, given the potential consequences to healthcare. Such research is highly needed to bridge the knowledge gap surrounding AI Chatbot integration in healthcare and provide insights to inform future interventions and policy development.

The recent Artificial Intelligence Index Report 2023 from Stamford University about the world countries’ acceptability ranked Saudi Arabia as one of the top three countries with a positive perception of AI products and services [10]. This study aims to further investigate these views among HCWs in Saudi Arabia and assess their perceptions of the Chatbot’s utility, identifying potential challenges in adopting AI Chatbots in healthcare and exploring factors influencing their usage, all within three months of ChatGPT’s global debut.

## 2. Methods

### 2.1. Study Design

This is a cross-sectional survey-based study. The survey questionnaire was developed and conducted by our multidisciplinary research team, comprising a medical informatics expert, a community medicine physician, a pediatric nephrologist, an adult infectious disease, a pediatric intensivist, a quality consultant, an adult intensivist, and a statistician. Their diverse backgrounds and expertise contributed to the design and development of the survey tool. The survey tool was developed in English based on a comprehensive PubMed search focusing on studies related to ChatGPT or similar AI innovations. Using the focus group technique, we then drafted and refined the survey, based on expert opinions from the research team in terms of content validity and relevance.

The survey was piloted among a group of 20 HCWs with diverse demographic backgrounds (8 physicians, 7 medical interns and students, and 5 nurses) to ensure its clarity, consistency, and suitability for the target population. As a result of their feedback, it was further refined to eliminate any ambiguities, ultimately resulting in a well-structured, valid, and reliable instrument for assessing HCWs’ perceptions of ChatGPT (Supplementary Materials). The survey is comprised of three parts. In the first part, HCWs responded to questions regarding their knowledge of ChatGPT nature and its usage for healthcare purposes. HCWs who indicated previous usage of ChatGPT were further asked regarding their satisfaction, expectations of its impact on the future of healthcare practice, and their potential purpose for using ChatGPT in medical practice. HCWs who did not use ChatGPT before the survey were specifically asked regarding their willingness to utilize it for healthcare purposes in the future.

The second part of the survey explored HCWs’ perceptions of any potential obstacles hindering the use of AI Chatbots in healthcare settings. The third and final part of the survey assessed the participants’ demographic and computer literacy characteristics as well as their potential to explore the ChatGPT further after completing the survey. 

The survey included multiple-choice questions, with respondents selecting one or more options to indicate their answers. No Likert scale was used in this study. For questions related to perceived obstacles of using AI in healthcare practice and the perceived usefulness of ChatGPT in healthcare practice, respondents were provided with multiple-answer options to select all applicable choices. No open-ended questions were included in the survey design.

### 2.2. Sampling Strategy and Participants Recruitment

The research team distributed the survey link electronically through HCWs’ social media channels in Saudi Arabia, over a period of 2 weeks (21 February–6 March 2023), using SurveyMonkey which has previously been utilized as an online platform for rapid deployment of electronic surveys among HCWs [11,12,13]. Inclusion criteria included HCWs in Saudi Arabia willing to participate in the study. Social media platforms used included Twitter and WhatsApp, email invitations, and personal contacts of the research team. This recruitment method was chosen to ensure broad geographic coverage and to include a diverse group of HCWs, considering the widespread use of these social media platforms among HCWs in Saudi Arabia [14].

### 2.3. Sample Size

Estimating a proportion of 50% of HCWs using ChatGPT and a margin of error of 0.05, confidence level of 95%, and study power of 80%, the sample size required for this study was 386. To account for incomplete and non-responses, the sample size was increased by 20%, making the minimum sample size required 463 HCWs.

### 2.4. Ethical Considerations

The Institutional Review Board (IRB) at King Saud University approved the study (Approval # 23/0155/IRB). Informed consent was obtained from all participants. Prior to participation in the survey, the purpose of the study was explained, and informed consent was obtained from participants on the first page of the electronic survey. Respondents had the opportunity to ask questions via the principal investigator’s email address. Personal identifiers were not collected to ensure confidentiality.

Role of the funding source: no funding source.

### 2.5. Statistical Analysis

Descriptive statistics: Categorically measured variables were analyzed using (frequencies with corresponding percentages). Data were tested for normality visually using histograms and Q-Q plots and statistically using the Kolmogorov–Smirnov statistical test of normality. The multiple response dichotomies analysis statistics were used for questions that accept multiple options (for example, obstacles hindering the use of AI Chatbots in healthcare settings). 

Regression analysis: multivariate-adjusted odds ratios (OR) and their 95% confidence intervals method were used to assess the association between dependent variables (for example, participants’ perception of ChatGPT impact on the future of healthcare practice) and participants’ characteristics. Statistical significance was measured using a two-tailed alpha test and set at the level of *p* < 0.05. Data were analyzed using the statistical package SPSS version 21 (IBM Corp., Armonk, NY, USA).

## 3. Results

A total of 1057 HCWs participated in the study. Most participants were male (57.4%), with the highest percentage belonging to the 25–34 years age group (39.5%), followed by 28.6% in the 18–24 age group. Almost half of the participants were physicians (48.8%), and 31.4% were medical interns or students. Approximately half of the participants (52.6%) had clinical experience of less than five years, while the remaining participants had more than five years of experience. Table 1 presents a detailed breakdown of the participants’ sociodemographic and professional characteristics.

**Figure 1:**
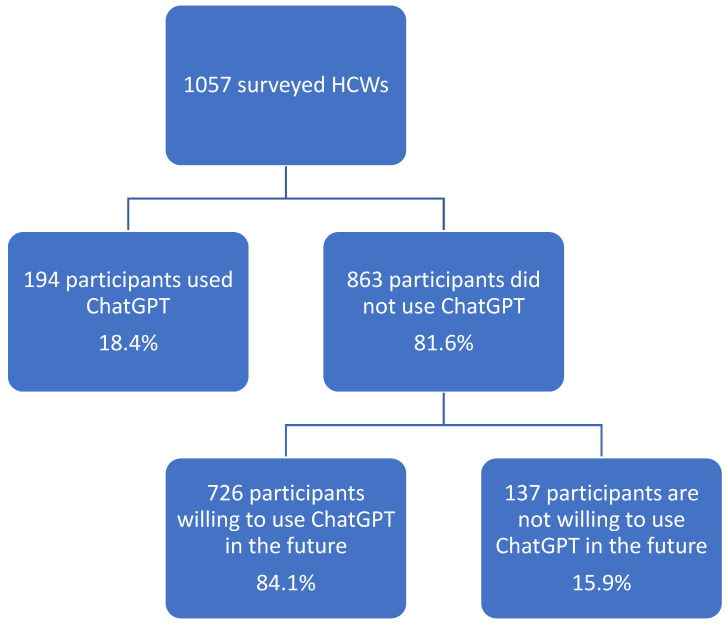
HCWs’ use of ChatGPT and expected future usage for healthcare purposes.

Most of the participants were familiar with computers and reported computer skills and expertise (44.3% familiar to some degree, 48.3% very familiar) (Table 2). As far as familiarity with ChatGPT is concerned, 50.9% were unfamiliar with ChatGPT; 15.1% were very familiar, and 34% reported being familiar to some degree. Among the surveyed HCWs, only 18.4% reported using ChatGPT for healthcare purposes as of the time of the survey, while 81.6% did not (Figure 1). Of those who did not use it, 84.1% expressed the expectation of using it in the future (Figure 1). Most participants expressed comfort using ChatGPT in their healthcare practice (61.9% were comfortable to some extent, and 13.2% were very comfortable) (Table 2). As a result of our survey, 81.8% of participants expressed a triggered interest in learning more about ChatGPT and other AI Chatbots.

Participants’ perceived usefulness of ChatGPT in healthcare practice were captured using the question with multiple choices and included providing medical decisions (39.5%), supporting patients and families (44.7%), appraising medical literature (48.5%), and aiding in medical research (65.9%) (Table 2).

As highlighted in Figure S1 (Supplementary Materials), HCWs in our study expect ChatGPT to have a significant impact on the future of healthcare practice. Most HCWs (76.7%) anticipate a positive effect; 17.7% anticipate a negative impact, and 5.6% anticipate no impact at all.

Table 3 displays HCWs’ perceived obstacles to using AI in healthcare practice. Most HCWs were concerned about the lack of credibility and unclear sources of information feeding AI Chatbots (46.9%), followed by concerns over harmful or incorrect medical decision recommendations made by AI models (40.2%). Over a third of HCWs (38.1%) expressed difficulty accessing AI models in their work setting, and 37.6% had concerns about AI models not being fully developed for medical practice use. Medicolegal implications associated with using AI in patient care were also a concern for many HCWs (30.8%). Interestingly 20.6% of HCWs were worried about AI models replacing human roles in healthcare practice (Table 3).

The Supplementary Materials with Table S1 shows the correlation between the HCWs’ expected credibility and their characteristics, while Table S2 identifies the factors correlating with their medicolegal concerns about the AI Chatbots use in healthcare practice as part of the obstacle question.

Table 4 summarizes HCW variables that correlated with their belief that ChatGPT has the potential to improve the future of the healthcare system. HCWs who believed that ChatGPT could improve patient outcomes had 7.92 times higher odds of believing that ChatGPT had the potential to improve the future of healthcare *p* < 0.001. This was followed by HCWs who reported being comfortable using ChatGPT for medical purposes (OR = 2.327, *p*-value < 0.001). Prior use of ChatGPT for healthcare purposes was associated with an increased positive expectation of ChatGPT improving the future of healthcare (OR = 1.902, *p* = 0.004). Similarly, the familiarity of HCWs with ChatGPT was associated with positive expectations for healthcare (OR = 2.023, *p*-value < 0.001). However, no correlation between HCWs’ trust in AI Chatbots or more specifically ChatGPT and the potential for ChatGPT to improve healthcare systems was found.

The variables associated with HCWs’ intention to use ChatGPT for healthcare purposes in the future are shown in Supplementary Materials Table S3, with their intention directly asked with only two choices: yes or no. HCWs were significantly more likely to use ChatGPT for healthcare purposes if they trusted in AI Chatbot capabilities for providing medical decisions (OR = 1.969, *p* < 0.001). Additionally, their previous use of ChatGPT correlated positively and significantly with their future intention to use it for healthcare purposes (OR = 2.601, *p* < 0.001). Participants who believed that ChatGPT has the potential to improve medical research and patients’ outcomes were significantly more inclined to use it in the future (OR = 1.835, *p* = 0.005, OR = 5.404, *p* < 0.001), respectively. Interestingly, HCWs who were concerned about AI Chatbots potentially taking over human roles in healthcare practice were significantly more inclined to use them for healthcare purposes in the future (OR = 1.969, *p* = 0.018). In contrast, the use of ChatGPT in the future was less likely to be considered by those who expressed concerns about the lack of personalized care provided by AI Chatbots (OR = 0.225, *p* = 0.003).

## 4. Discussion

### 4.1. Principal Results

With the new launch of ChatGPT, it is important to explore HCWs’ knowledge and skills as well as their viewpoints on the use of AI in general and of ChatGPT specifically. Such a study sheds light on the future studies needed in this field. Our study found that while only one-fifth of HCWs in our cohort used ChatGPT for medical purposes as of the survey time; still, about half of the respondents were familiar with ChatGPT. As the adoption of ChatGPT expands rapidly and extensively, both locally and internationally, a growing number of healthcare workers are expected to utilize AI Chatbots, which could directly or indirectly influence the dynamics of digital healthcare [15].

Initial research assessed various ChatGPT’s feasibility in healthcare, such as tasking it with composing patients’ medical note after providing information in a random order [16]. ChatGPT generated a structured note, correctly categorized most parameters, and suggested further therapies based on the provided information, even when the information was nonspecific. Furthermore, ChatGPT demonstrated that it is capable of passing the United States Medical Licensing Exam (USMLE) [17]. Recent findings revealed ChatGPT’s human-level performance on multiple medical examinations as well, such as the USMLE (60.2%), MedMCQA (57.5%), and PubMedQA (78.2%) [18]. With its unique “ChatGPT Improved Accuracy” (CGA) model, ChatGPT can outperform other Chatbots in terms of precision, coherence, and readability by learning from its mistakes and producing more accurate results [19].

Our study stands as a pioneering effort to determine the prevalence of ChatGPT users among HCWs, addressing a significant gap in the current literature. Although ChatGPT has experienced record-breaking subscription rates since its launch, becoming the fastest-growing app in history, there remains a paucity of data regarding its adoption and usage among HCWs. The current adoption rate of ChatGPT is remarkable when compared to other highly successful apps. For instance, Instagram took around 2.5 months to reach 1 million users (about the population of Delaware) in 2010, while Spotify required nearly six months [15]. In contrast, ChatGPT amassed 1 million users within just five days and achieved 100 million users in a mere two months [15]. About three-quarters of our surveyed HCWs expressed comfort using ChatGPT and expected future positive impact on the future of the healthcare system owing to it. Our results stress the need to introduce HCWs to AI Chatbots including ChatGPT and educate them on the use of such platforms. This is especially important with the recent widespread AI Chatbots platform in all sectors of science and knowledge, including the healthcare setting [20].

HCWs who expressed significant trust in AI Chatbots or believed in their promising impact on patients’ outcomes and medical research showed significant interest in using it in the future in healthcare practice. The expectation of the positive impact of ChatGPT on the future of healthcare correlated with their familiarity and previous use of it, their comfort using it for medical purposes, and their belief in its potential improvement of patients’ outcomes.

Medicolegal concerns about using AI Chatbots in medical practice were the most common obstacle identified by the participating HCWs, especially among physicians. Additionally, those who had questioned the credibility of AI Chatbots or worried about breaking patient’s confidentiality or recommending harmful or wrong medical decisions and expectations of HCWs’ resistance to adopting AI Chatbots all were associated with high medicolegal concerns.

### 4.2. Perceived Usefulness of ChatGPT

The majority of HCWs expressed a desire to use ChatGPT in the future. Although there is still some uncertainty about the impact of ChatGPT on the healthcare setting [21], the findings from this study show that ChatGPT was looked at as having the potential to become a valuable tool in healthcare. This is based on the HCWs’ perception of the tool being a useful resource in supporting patient care, medical research, and appraisal of medical literature. Interestingly, this positive perception aligns with the broader sentiment toward AI in Saudi Arabia. According to the Artificial Intelligence Index Report 2023 by Stanford University, 76% of Saudi respondents agreed that products and services using AI have more benefits than drawbacks, making it the second most positive country towards AI after China [10]. This suggests a favorable environment for the adoption and integration of AI tools like ChatGPT in the Saudi healthcare system.

The high level of interest generated by our study indicates that HCWs are eager to learn more about ChatGPT and other AI Chatbots. ChatGPT was frequently utilized to quickly generate educational materials and provide healthcare advice to patients and communities [22,23,24]. Therefore, providing more educational resources and training programs on AI Chatbots could enhance their usability and usefulness in healthcare. Prior to the safe integration of these tools into healthcare practice, it is imperative to conduct research and develop robust oversight mechanisms to guarantee their accuracy and dependability [25].

### 4.3. Trust and Credibility of ChatGPT

The current study revealed that HCWs showed moderate to low levels of trust in ChatGPT’s ability to generate medical decisions, and only a small proportion showed a high level of trust. These findings are consistent with previous studies that have reported concerns about the accuracy and reliability of AI Chatbots in healthcare and were labeled as “Artificial Hallucinations” [26,27]. Howard et al. suggested that the major obstacle to the adoption of ChatGPT in healthcare settings was deficiencies in situational awareness, inference, and consistency [26].

HCWs users of ChatGPT and those with more clinical experience, self-rated familiarity, and comfort level in using ChatGPT for medical purposes a positive perception of its usefulness and with higher levels of trust. Therefore, providing more evidence-based data on ChatGPT’s accuracy and reliability in healthcare could help enhance HCWs’ trust in its ability to generate medical decisions [28]. ChatGPT suggestions could complement the optimization of clinical decision support alerts, assist in identifying potential improvements, and offer unique perspectives [29]. The suggestions generated by AI were thought to be highly understandable and relevant with moderate usefulness but with low acceptance, bias, inversion, and redundancy [30].

### 4.4. Obstacles and Concerns about ChatGPT

Our study identified several obstacles and concerns about ChatGPT’s use in healthcare, including its lack of credibility and questioned source of information, medicolegal implications, resistance to its use, and concerns about patient confidentiality and personalized care. However, the most significant obstacle identified was ChatGPT’s lack of credibility and question of the source of information, which is consistent with previous studies that have reported similar concerns about AI Chatbots’ accuracy and reliability [26,27]. 

### 4.5. Medicolegal Implications of ChatGPT

HCWs perceived medicolegal implications as a significant barrier to ChatGPT’s use in healthcare. Physicians were significantly more likely to perceive medicolegal concerns, which could be due to their greater awareness of legal and ethical issues in healthcare. One study showed that physicians had a deeper level of specific training in ethics than nurses [30]. Familiarity with ChatGPT correlated positively with HCWs’ concerns, indicating that those who were more familiar with ChatGPT may be more aware of its potential risks and limitations.

However, previous use of ChatGPT was associated with fewer medicolegal concerns in this study. Besides abiding by the local bylaws that regulate the use of AI in medical practice, HCWs and society should ensure that the AI model being used is “trustworthy” [31]. The European Commission has outlined a checklist for trustworthy AI, which includes requirements for human oversight, robustness and safety, data privacy, process transparency, equitability, societal well-being, and accountability [32]. The checklist emphasizes the need for algorithms and data to be auditable and accessible and for redressal processes to be fair and equally accessible [31].

Notably, HCWs expressed concerns regarding the credibility of AI Chatbots and their potential medicolegal implications. These concerns were positively associated with questioning the credibility of AI Chatbots and a lack of comfort in using them in medical practice [33]. The perceived medicolegal implications of using AI Chatbots were higher among physicians and those with more experience, indicating the need for clear guidelines and regulations around the use of AI Chatbots in healthcare. A recent study found that transparency, accountability, and user-centered design are key factors that can improve the acceptance of AI Chatbots among healthcare professionals [34]. Furthermore, it is essential to ensure that AI Chatbots are designed with appropriate safeguards to prevent misuse of patient data, protect privacy, and maintain the trust of healthcare workers. The integration of AI Chatbots into healthcare systems requires not only technological advancements but also the establishment of ethical, legal, and social frameworks to address the potential challenges [32,34].

Overall, the results of this study highlight HCWs’ perception of ChatGPT in the context of healthcare. While most participants showed a lack of familiarity with ChatGPT, the majority of those who had not used it expressed an interest in using it in the future, and most participants felt comfortable using it in their healthcare practice. This indicates that while there is a need for further education and training, healthcare workers are open to using AI Chatbots like ChatGPT in their practice. Prior to the ChatGPT era, several papers described adaptations of AI Chatbots by specific medical disciplines, with promising initial results [35]. Furthermore, the evolution of newer generations of AI Chatbots, such as ChatGPT-4, warrants vigilant adaptations of their enhanced features [36,37].

It is also important to note that while HCWs expressed positive expectations for ChatGPT’s impact on the future of healthcare, they also expressed concerns about the potential for AI Chatbots to take over human roles in healthcare and the lack of personalized care when using them. These concerns suggest that while AI Chatbots have the potential to alter healthcare outcomes, they should be used in conjunction with human care and should not replace it. This should be among the research priorities soon, as we expect this AI Chatbot and healthcare deliveries to interact together at exponential steps [28].

### 4.6. Limitations

This study has several limitations and strengths that should be considered when interpreting the results. As a cross-sectional survey study, this research is subject to some limitations, including sampling bias, response bias, and recall bias. The sampling technique may lead to selection bias, as the participants may not be representative of the entire population of healthcare workers. Response bias may occur if participants provide answers that are socially desirable, if they misunderstand the questions, or if there were other unmeasured confounding factors that could have influenced the results. For instance, almost half of the study sample reported unfamiliarity with ChatGPT, which could introduce a source of bias that must be considered. The unfamiliarity with ChatGPT could be viewed from multiple perspectives such as the expected time for technology to penetrate the community, variables related to the acceptability of new changes, the impact of age and variable generations’ features, and the ethical and legal regulation related to the adoption of new technology.

Recall bias may influence the results if the participants have difficulty remembering their experiences with ChatGPT; however, ChatGPT was launched only three months prior to our study, and about half of our HCWs were not yet familiar with it.

Additionally, the anonymity of the survey responses will ensure that participants feel comfortable providing honest and accurate responses. So, another limitation of our study is that we did not collect specific regional information from the respondents due to privacy concerns. Therefore, while respondents came from different regions of Saudi Arabia, we were unable to provide detailed information about the representation of each specific region. This lack of granularity regarding regional representation should be taken into consideration when interpreting the findings. Considering the absence of data on the total population size from which the sample was drawn, the generalizability of the study findings to the broader population may be limited. The study included respondents from one country and a larger multi-countries study is warranted.

We also acknowledge the importance of further research to investigate and demonstrate the potential benefits and mechanisms of ChatGPT or other AI Chatbots’ impact on healthcare practice. As our study did not include qualitative techniques, we suggest that future research inclusion of such methods could provide additional insights and a more comprehensive understanding of HCWs’ perspectives on ChatGPT, such as exploring the medicolegal implications in greater depth. This could be a valuable direction for future studies in this field. By conducting more comprehensive research, including in-depth investigations into the concerns, accuracy, reliability, and medicolegal implications associated with AI Chatbots, we can explore more insights and recommendations to address these issues. This will not only contribute to the advancement of AI technology in healthcare but may also enhance patient care and outcomes. Furthermore, future research should also aim to compare ChatGPT with other AI-based Chatbots and traditional medical decision-making methods. Additionally, we suggest conducting studies that may assist policymakers to evaluate the actual performance of AI Chatbots to establish standards and guidelines for their accuracy and reliability and implement appropriate quality assurance mechanisms to gain greater insights into AI Chatbots and promote their responsible use in healthcare settings.

### 4.7. Comparison with Prior Work

Despite these limitations, this study has several strengths. To the best of our knowledge, this is the first study that explored early HCWs’ usage of ChatGPT. Additionally, the study has a relatively large sample size of HCWs from different regions in Saudi Arabia. The study will provide valuable insights into the perceptions of HCWs about ChatGPT, including their knowledge, attitude, obstacles, and intended practice, for future research and stakeholders.

## 5. Conclusions

This study provides insights into HCWs’ perceptions of ChatGPT within the first three months of its launch. While ChatGPT is seen as a potentially beneficial tool in healthcare settings, concerns about its accuracy, reliability, and medicolegal implications persist. Addressing these concerns and ensuring the trustworthiness and dependability of AI Chatbots is essential for promoting their adoption in healthcare settings. Although the reported unfamiliarity with ChatGPT needs to be considered as a potential source of bias, it is a significant trigger for research exploring this issue. Furthermore, the study findings underscore the importance of further research to evaluate the clinical outcomes associated with ChatGPT and to benchmark its effectiveness against other AI Chatbots in healthcare applications. Future studies should also consider exploring the impact of ChatGPT on healthcare practice among both familiar and unfamiliar users to gain a more comprehensive understanding of its potential benefits and limitations. Additionally, providing actionable strategies to address concerns about accuracy and reliability may contribute to more responsible use of AI Chatbots in healthcare.

## Figures and Tables

**Table 1:** Healthcare workers’ sociodemographic and professional characteristics. N = 1057.

	Frequency	Percentage
**Sex**		
Female	450	42.6
Male	607	57.4
**Age group**		
18–24 years	302	28.6
25–34 years	418	39.5
35–44 years	170	16.1
45–54 years	105	9.9
55–64 years	62	5.9
**Clinical Role**		
Physician	516	48.8
Medical Interns and students	332	31.4
Nurse	139	13.2
Technicians, therapists, and pharmacists	70	6.6
**Healthcare experience**		
<5 years	556	52.6
5–10 years	187	17.7
10–20 years	178	16.8
>20 years	136	12.9

**Table 2:** Healthcare workers’ perceptions of ChatGPT and artificial intelligence models. N = 1057.

	Frequency	Percentage
**Participants’ computer skills/expertise**		
Not so familiar	78	7.4
Familiar to some degree	468	44.3
Very familiar	511	48.3
**How familiar are you with the term “ChatGPT”?**		
Not so familiar	538	50.9
Familiar to some degree	359	34
Very familiar	160	15.1
How comfortable would you be using ChatGPT in your healthcare practice?		
Not comfortable at all	263	24.9
Comfortable to some extent	654	61.9
Very Comfortable	140	13.2
**Did this survey raise your interest to read about ChatGPT and other AI models?**		
No	192	18.2
Yes	865	81.8
**Participants’ perception of ChatGPT usefulness in healthcare practice? (Selection can be multiple choices)**		
Providing medical decisions	418	39.5
Providing support to patients and families	473	44.7
Provide an appraisal of medical literature	513	48.5
Medical research aid (like drafting manuscripts)	697	65.9

**Table 3:** HCWs’ perceived obstacles to using AI (Artificial Intelligence) in healthcare practice currently.

	Frequency	Percentage
Lack of credibility/Unknown source of information of data in the AI Model	496	46.9
Worry of harmful or wrong medical decisions recommendations	425	40.2
Not available in my setting	403	38.1
AI Chatbots are not yet well-developed	397	37.6
Medicolegal implications of using AI for patients’ care	326	30.8
I do not know which AI model can be used in healthcare	311	29.4
Unfamiliarity with using AI Chatbots	296	28.0
Worry about patient’s confidentiality	273	25.8
Resistance to adopt AI Chatbot in medical decisions	249	23.6
Worry of AI taking over human role in healthcare practice	218	20.6
Others (lack of personalized care and inability to adapt to prognostic factors)	43	4.1

**Table 4:** Multivariate binary logistic regression analysis of healthcare workers’ variables and their expectation of ChatGPT potential improvement of the healthcare future (N = 1057).

Variable?	Multivariate Adjusted Odds Ratio	OR 95% C.I.		*p*-Value
		Lower	Upper	
Sex	0.772	0.536	1.112	0.164
Age	0.960	0.822	1.122	0.609
Clinical role	1.048	0.858	1.281	0.646
Trust *	0.804	0.627	1.031	0.085
History of ChatGPT use at the time of the survey	1.902	1.226	2.950	0.004
Familiarity with ChatGPT	2.023	1.508	2.714	<0.001
Medical decisions ^@^	1.463	0.994	2.154	0.054
Comfort level ^Ψ^	2.327	1.650	3.281	<0.001
Patients’ outcomes ^Σ^	7.927	5.046	12.452	<0.001
Constant	0.006			<0.001

Dependent variable = Participants’ expectation of potential healthcare future improvement by ChatGPT. * Trust level in AI’s ability to provide medical decisions for healthcare providers; **@** Belief in ChatGPT’s ability to provide valuable medical decisions; ^Ψ^ Using ChatGPT for medical purposes/practice comfort level; **^Σ^** Belief of ChatGPT patients’ outcomes improvement.

## Data Availability

The deidentified participant data collected for this study will be made available to others after 30 November 2023, upon reasonable request from the corresponding author, with investigator support, after approval of a proposal, in agreement with the IRB-provided signed data sharing agreement.

## Supplementary Materials

The following supporting information can be downloaded at: https://www.mdpi.com/article/10.3390/healthcare11131812/s1, Figure S1: Healthcare workers’ perceptions of ChatGPT impact on the future of healthcare system: title; Figure S2: Participants’ conception of ChatGPT; Figure S3: Participants’ trust of ChatGPT provide medical decisions for HCWs; Table S1: Multivariate Binary Logistic Regression Analysis of healthcare workers’ showing predictors associated with the trust of AI Chatbots credibility; Table S2: Multivariate Logistic Binary Regression analysis of healthcare workers’ variables and their worry of arising medicolegal concerns of AI Chatbot use for patient care; Table S3: Multivariate Binary Logistic Regression of healthcare workers’ variables and their intention of ChatGPT use in the future for healthcare purposes.

## Abbreviations

AI	Artificial Intelligence
CGA	ChatGPT Improved Accuracy
ChatGPT	Chat Generative Pre-trained Transformer
HCWs	healthcare workers
USMLE	United States Medical Licensing Exam
