# Supplementary material for: ChatGPT and the Future of Digital Health: A Study on Healthcare Workers’ Perceptions and Expectations
Source: Healthcare (Basel). 2023 Jun 21;11(13):1812. doi: 10.3390/healthcare11131812 (PMC10340744; doi:10.3390/healthcare11131812)
Supplement: Supplementary file 1 [file healthcare-11-01812-s001.zip › healthcare-2392171-supplementary.pdf]

Supplementary Materials:

Table of contents:

Figure S1: Healthcare workers' perceptions of ChatGPT impact on the future of the healthcare system (Page 2)

Figure S2: Participants' conception of ChatGPT (Page 3)

Figure S3: Participants' trust of ChatGPT provides medical decisions for HCWs (Page 4)

Table S1: Multivariate Binary Logistic Regression Analysis of healthcare workers' showing predictors associated with the trust of AI chatbot credibility. (Page 5)

Table S2: Multivariate Logistic Binary Regression analysis of healthcare workers' variables and their worry of arising medicolegal concerns of AI Chatbot use for patient care. (Page 6)

Table S3: Multivariate Binary Logistic Regression of healthcare workers' variables and their intention of ChatGPT use in the future for healthcare purposes. (Page 7)

Questionnaire tool. (Pages 8-9)

Figure S1: Healthcare workers' perceptions of ChatGPT impact on the future of the healthcare system (N=1057)

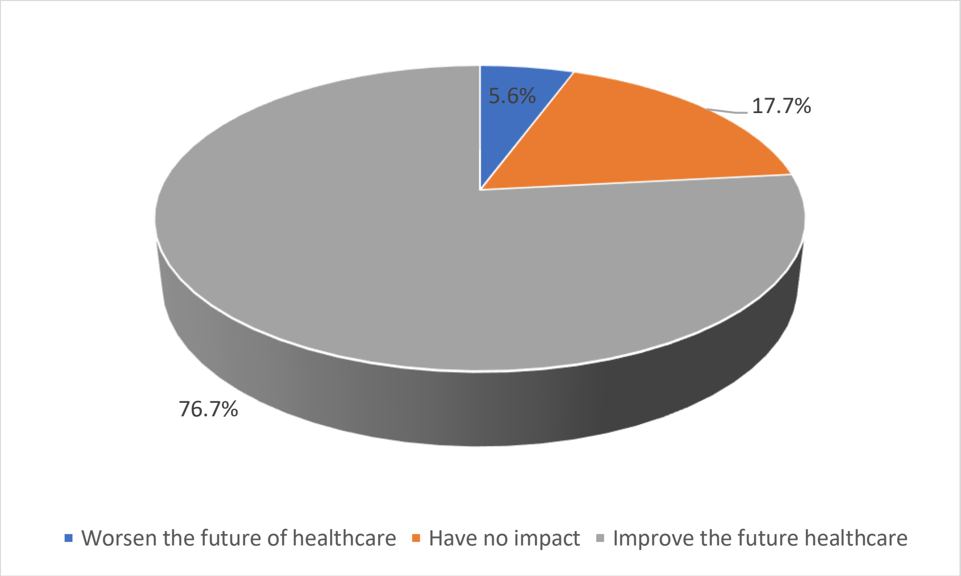

Figure S2: Participants' conception of ChatGPT

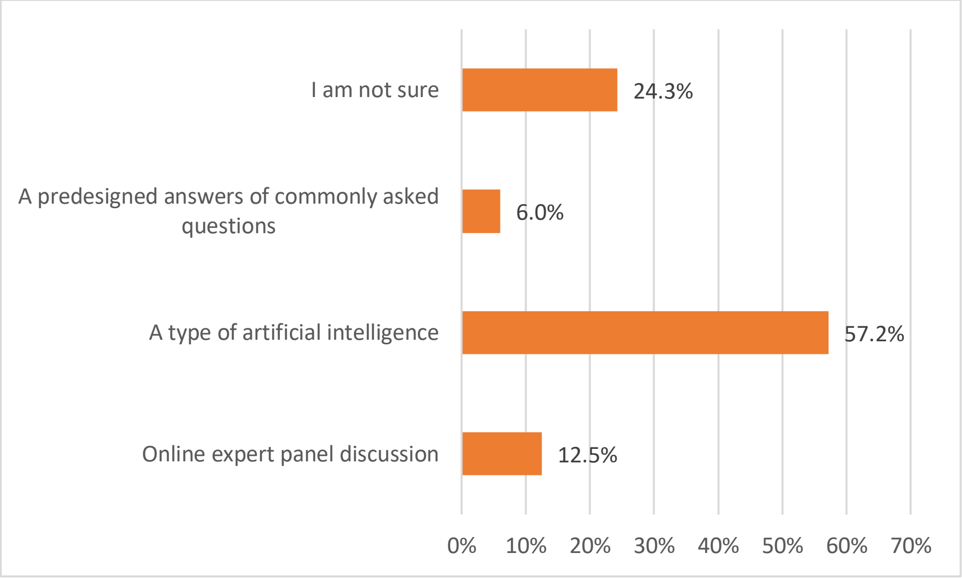

57.2% of the participants correctly answered that ChatGPT is a type of artificial intelligence, 24.3% were not sure of its nature, 12.5% thought it is an online expert panel discussion platform, and 6.0% felt a preset of predesigned answers for commonly asked questions.

Figure S3: Participants' trust of ChatGPT provides medical decisions for HCWs

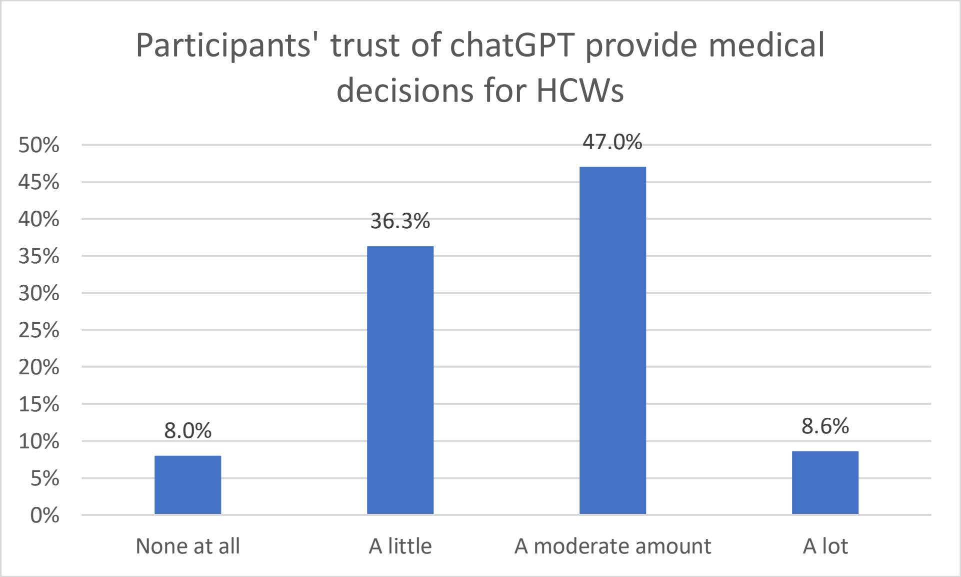

47% of the HCWs showed moderate trust while 36.3% showed little trust, and only 8.6% showed a high trust in ChatGPT generating medical decisions

Table S1: Multivariate Binary Logistic Regression Analysis of healthcare workers' showing predictors associated with the trust of AI chatbots credibility. N=1057

Variable	Multivariate adjusted Odds Ratio	OR 95% C.I.		p-value
		Lower	Upper	
Sex=Male	1.050	0.780	1.414	0.749
Age group >=35 years	0.674	0.495	0.918	0.012
Clinical Role	0.948	0.811	1.109	0.507
Participants' Trust in AI Chatbots to provide medical decisions	0.749	0.616	0.911	0.004
Used ChatGPT at the time of the survey	0.540	0.371	0.786	0.001
The belief of AI chatbots benefit medical research	1.462	1.078	1.983	0.015
Believes that AI Chatbots are not yet well-developed	1.766	1.312	2.377	<0.001
Worry about breaking patient's confidentiality within AI chatbots	1.403	1.005	1.957	0.047
Worry about AI chatbots recommendation of harmful or wrong medical decisions	3.145	2.336	4.234	<0.001
Worry about Medicolegal implications of using AI chatbots	1.781	1.295	2.450	<0.001
Other concerns about using AI chatbots (lack of personalized care and inability to adapt to prognostic factors)	0.266	0.128	0.551	<0.001
Awareness that ChatGPT is a type of artificial Intelligence interface	2.648	1.957	3.583	<0.001
Constant	0.673			0.268

Dependent variable = Participants' trust in AI chatbots' credibility

Table S1 shows the correlation between credibility and HCWs' variables. HCWs aged 35 years and above were less likely to question the credibility (OR .674, p-value=0.012). Participants who correctly identified ChatGPT as an AI platform had high concerns about AI Chatbots' credibility in general (OR=2.648, p-value<0.001). While HCWs' trust in the AI chatbots' ability to produce medical decisions, and those who had a history of using ChatGPT had low concerns about AI Chatbots' credibility (OR=0.749, p-value=0.004, OR=0.540, p-value=0.001) respectively. While HCWs' belief of AI chatbot's ability to help in medical research, their belief that they are not yet well-developed, concerns about patient confidentiality and concerns about AI Chatbots recommending harmful or wrong medical decisions or raising medicolegal concerns correlated significantly with high concerns of AI Chatbots credibility (OR=1.462, p-value=0.015, OR=1.766, p-value<0.001, OR=1.403, p-value=0.047, OR=3.145, p-value<0.001, OR=1.781, p-value<0.001,) respectively. Conversely, HCWs who had other minor concerns of AI Chatbot use were less likely to question their credibility (OR=0.266, p-value<0.001).

Table S2: Multivariate Logistic Binary Regression analysis of healthcare workers' variables and their worry about arising medicolegal concerns of AI Chatbot use for patient care. N=1057

**Variable**	**Multivariate adjusted Odds Ratio**	**OR 95% C.I.**		**p-value**
		**Lower**	**Upper**	
Sex=Male	1.041	0.762	1.422	0.801
Age group	1.069	0.923	1.239	0.374
Profession=Physician	1.469	1.050	2.055	0.025
Familiarity with ChatGPT	1.253	1.004	1.563	0.046
History of ChatGPT use at the time of the survey	0.562	0.389	0.811	0.002
The belief of AI Chatbots questioned the credibility	1.772	1.288	2.437	<0.001
Worry of breaking patient's confidentiality within AI chatbots	2.215	1.614	3.040	<0.001
Worry about AI Chatbots recommending harmful or wrong medical decisions	1.882	1.374	2.578	<0.001
HCWs' unfamiliarity with AI chatbots as a barrier to their use in healthcare practice	1.416	1.021	1.964	0.037
HCWs' resistance to AI chatbots as a barrier to their use in healthcare practice	3.018	2.171	4.195	<0.001
Belief in ChatGPT helps with medical literature critical appraisal	1.396	1.033	1.886	0.030
Constant	0.105			<0.001

Dependent variable= Participants' worry about arising medicolegal concerns of AI Chatbot use for patient care

Table S2 presents HCWs' variables associated with their medicolegal concerns. Physicians were significantly more likely to have medicolegal concerns (OR=1.469, p-value=0.025). Familiarity with ChatGPT also correlated

significantly and positively with their concerns (OR=1.253, p-value=0.046). While previous ChatGPT use was associated significantly with fewer concerns (OR=0.562, p-value=0.002). The belief of AI Chatbots questioned credibility, concerns about patients' confidentiality, and worry of recommending harmful decisions, all correlated significantly and positively with HCWs' medicolegal concerns about using ChatGPT in medical practice (OR=1.772, p-value<0.001, OR= 2.215, p-value<0.001, OR 1.882, p-value<0.001) respectively. HCWs' perceived barriers to using AI Chatbots like, such as unfamiliarity and resistance to its use, and their belief of ChatGPT's potential use in medical literature critical appraisal correlated significantly and positively with high medicolegal concerns (OR=1.416, p-value=0.037, OR=3.018, p-value<0.001, OR=1.396, p-value=0.030) respectively.

Table S3: Multivariate Binary Logistic Regression of healthcare workers' variables and their intention of ChatGPT use in the future for healthcare purposes, n=863.

Variable	Multivariate adjusted Odds Ratio	OR 95% C.I.		p-value
		Lower	Upper	
Sex=Male	0.735	0.476	1.136	0.166
Age group	1.134	0.926	1.389	0.224
Clinical Role	0.891	0.703	1.131	0.344
Trust*	1.969	1.467	2.644	<0.001
ChatGPT use at the time of the survey	2.601	1.621	4.175	<0.001
Worry#	1.969	1.126	3.444	0.018
Other concerns about using AI chatbots$	0.225	0.085	0.598	0.003
Medical research$^{\Omega}$	1.835	1.201	2.805	0.005
Patients' outcomes$^{\Sigma}$	5.404	3.522	8.293	<0.001
Constant	0.132			<0.001

Dependent variable = Participants' intention of ChatGPT use in the future for healthcare purposes

***** Trust level in AI's ability to provide medical decisions for healthcare providers

Worry about AI Chatbots' potential to take over the human role in healthcare

$(lack of personalized care and inability to adapt to prognostic factors)

Ω Belief of ChatGPT benefit in medical research

$^{\Sigma}$ Belief in ChatGPT patients' outcomes improvement

Dear healthcare colleague,

You are invited to participate in this survey, even if you did not use ChatGPT before, and your contribution is greatly valued. At the end of the survey, you will be redirected to a YouTube video on ChatGPT.

Study Title: ChatGPT Knowledge, Attitude, and Intended Practice among Healthcare Workers: A Cross-sectional survey

The study is approved by IRB at King Saud University (23/0155/IRB).
The purpose of this online survey is to explore the HCWs' KAP in regard to the AI model ChatGPT. It will take approximately 2-3 minutes of your time. Your participation is completely voluntary.

The responses are completely anonymous and confidential, and researchers will not be able to identify participants. We will use this collected information for research purposes only.

Please feel free to call 0114692002 to answer your questions.

AGREEMENT ON PARTICIPATION IN THIS STUDY:
If you are willing to participate in this online questionnaire-based survey, please continue the survey.

Thank you!

On behalf of the Research Team:
Prof Hani Temsah, Prof Ayman Al-Eyadhy, Prof Amr Jamal

* 1. How familiar are you with the term "ChatGPT"?
Very familiar
Somewhat familiar
Not familiar at all
* 2. What do you think ChatGPT is?
Online expert panel discussion
A type of artificial intelligence
A predesigned answer to commonly asked questions
I am not sure
* 3. How do you think ChatGPT can be useful in healthcare?
(Please choose all that apply)
Providing medical decisions
Providing support to patients and families
Provide an appraisal of medical literature
Helping in medical research (like drafting manuscripts)
* 4. How comfortable would you be using ChatGPT in your healthcare practice?
Very comfortable
Somewhat comfortable
Not comfortable at all
* 5. Do you think ChatGPT can improve patient outcomes?
Agree
Neither agree nor disagree
Disagree
* 6. How do you think ChatGPT can impact the future of healthcare?
Improve the future of healthcare
Have no impact
Worsen the future of healthcare
* 7. Have you used ChatGPT before?
Yes
No

How was your ChatGPT experience?

* 8. If you have used ChatGPT in your healthcare practice, how satisfied were you with the experience?
Satisfied

Neutral
Dissatisfied

* 9. As you have not used ChatGPT in your healthcare practice until now: would you consider using it in the future?
Yes
No

General Questions:

* 10. What do you think are the main obstacles to using AI (Artificial Intelligence) in healthcare at this time?
AI Chatbots are not yet well-developed
Not available in my setting
Lack of credibility / Unknown source of information that feeds the data to the AI Model
I do not know which AI model can be used in healthcare
I worry about my patient's confidentiality if I use AI in their treatment
I am worried AI will take over the human roles in healthcare
I am worried it may recommend harmful or wrong medical decisions
Unfamiliarity with using AI Chatbots
Resistance of some healthcare providers to adopt AI Chatbot in medical decisions
Medicolegal implications of using AI for my patients' care
Other (please specify)

* 11. How much do you describe your computer knowledge/expertise?
Very familiar
Somewhat familiar
Not so familiar

* 12. What is your profession?
Physician
Intern
Medical student
Nurse
Other healthcare providers (please specify)

* 13. How many years of experience do you have in healthcare?
Less than 5 years
5-10 years
10-20 years
More than 20 years

* 14. What is your gender?
Female
Male

* 15. What is your age
Under 18
18-24
25-34
35-44
45-54
55-64
65+

* 16. How much do you trust AI (Artificial Intelligence) in producing medical decisions for healthcare providers?
A lot
A moderate amount
A little
None at all

* 17. Did this survey make you more interested to read about ChatGPT and other AI models?
Yes
No

18. In what country do you currently reside?
Saudi Arabia
Other (please specify)
